# Table in the corner: a qualitative study of life situation and perspectives of the everyday lives of oesophageal cancer patients in palliative care

**DOI:** 10.1186/s12904-019-0445-2

**Published:** 2019-07-22

**Authors:** Louise Laursen, Mai Nanna Schønau, Heidi Maria Bergenholtz, Mette Siemsen, Merete Christensen, Malene Missel

**Affiliations:** 1 Department of Palliative Care, Copenhagen University Hospital, Copenhagen, Denmark; 2 Department of Cardiothoracic Surgery, Copenhagen University Hospital, Rigshospitalet, Blegdamsvej 9, 2100 Copenhagen, Denmark; 3 Roskilde University, Roskilde, Denmark; 4 Medical and Surgical Department, Holbaek Sygehus, Denmark & The Danish Knowledge Centre for Rehabilitation and Palliative Care, University of Southern Denmark, Odense, Denmark

**Keywords:** Oesophageal cancer, Palliative care, Qualitative research, Patient perspective, Human dignity

## Abstract

**Background:**

Incurable oesophageal cancer patients are often affected by existential distress and deterioration of quality of life. Knowledge about the life situation of this patient group is important to provide relevant palliative care and support. The purpose of this study is to illuminate the ways in which incurable oesophageal cancer disrupts the patients’ lives and how the patients experience and adapt to life with the disease.

**Methods:**

Seventeen patients receiving palliative care for oesophageal cancer were interviewed 1–23 months after diagnosis. The epistemological approach was inspired by phenomenology and hermeneutics, and the method of data collection, analysis and interpretation consisted of individual qualitative interviews and meaning condensation, inspired by Kvale and Brinkmann.

**Results:**

The study reveals how patients with incurable oesophageal cancer experience metaphorically to end up at a “table in the corner”. The patients experience loss of dignity, identity and community. The study illuminated how illness and symptoms impact and control daily life and social relations, described under these subheadings: “sense of isolation”; “being in a zombie-like state”; “one day at a time”; and “at sea”. Patients feel alone with the threat to their lives and everyday existence; they feel isolated due to the inhibiting symptoms of their illness, anxiety, worry and daily losses and challenges.

**Conclusions:**

The patients’ lives are turned upside down, and they experience loss of health, function and familiar, daily habits. The prominent issues for the patients are loneliness and lack of continuity. As far as their normal everyday lives, social networks and the health system are concerned, patients feel they have been banished to a “table in the corner”. These patients have a particular need for healthcare professionals who are dedicated to identifying what can be done to support the patients in their everyday lives, preserve dignity and provide additional palliative care.

## Background

Oesophageal cancer is a significant cause of all cancer-related deaths worldwide with its aggressive nature, early spread, rapid tumour recurrence, and poor prognosis [1, 2]. In order to present the oesophageal cancer patient population it is known, that there is a significant difference between gender distributions; the incidence of this disease is about 2–4 fold higher among males compared to females [2, 3]. Two histologic types account for the majority of oesophageal cancers; adenocarcinoma and squamous cell carcinoma. The epidemiology of these types varies markedly [3, 4]. Squamous cell carcinoma (SCC) is the most common type of cancer in the upper part of the oesophagus and smoking and alcohol are major risk factors for developing SCC [1–3]. Additionally, SCC are correlated with low socio-economic status [5, 6].

Once symptoms are present (e.g., dysphagia, in most cases), oesophageal cancers have usually spread beyond the oesophagus [1, 4]. In such cases, surgery is not an option and palliative treatment is offered in the form of chemotherapy, radiotherapy or local treatment using dilatation and/or oesophageal stent. Palliative treatment aims to control disease-related symptoms, preserve as good a quality of life as possible, and prolong survival. Median survival for patients with advanced oesophageal cancer without treatment is less than 6 months [2]. Chemotherapy improves survival, but the survival benefit is limited and must be weighed against side-effects of treatment. Oesophageal stenting may offer relief of dysphagia however, survival is not associated with whether the stent is covered or not [2, 7]. Palliative care can be delivered on different levels, and usually a three-level approach of palliative care can be found: 1) a palliative care approach which is used in settings and services only occasionally treating palliative care patients. All professionals working in healthcare should be confident with the basic palliative care principles and able to put them into practice; 2) general palliative care provided by primary care professionals and specialists treating patients with life-threatening diseases who have good basic palliative care skills and knowledge; and 3) specialist palliative care provided by specialised services for patients with complex problems not adequately covered by other treatment options. Specialist palliative care describes services whose main activity is the provision of palliative care which is requiring a higher level of education, staff and other resources [8, 9]. The patients with SCC typically receive treatment in the cardiothoracic surgery or oncology departments, which are not part of the specialised palliative care setting, and thus their palliative needs might be overseen [10]. It is reported that only half of the patients admitted to a general palliative care setting receive the palliative care they need [11, 12].

There exist few qualitative studies about how oesophageal cancer affects patients and their everyday life. Studies have described how living with incurable oesophageal cancer can give rise to emotional distress and fear and how the illness can trigger anxiety, chaos, insecurity and a lower quality of life [13–15]. We need more knowledge about the life situation of these patients to meet their need for support through the course of the illness and treatment and thereby be able to develop supporting, palliative care interventions based on the patients’ perspective to complement the treatment. This knowledge is particularly needed within the general palliative care setting, which has a tendency to distinguish sharply between curative and palliative care and treatment, often in a fragmented way [16, 17]. According to the World Health Organization, palliative approaches are about more than just controlling symptoms [18], and studies show that patients with incurable oesophageal cancer need more than objective assessments from the hospital system [14]. Many studies point out that research addressing the social and psychological needs of patients suffering from a physical illness lags behind studies of the physical aspects of illness [19, 20]. The purpose of this study is to illuminate the ways in which incurable oesophageal cancer disrupts the patients’ lives and how the patients experience and adapt to life with the disease in order to suggest palliative care interventions.

## Methods

The study was designed as a qualitative investigation of patients undergoing palliative treatment for oesophageal cancer.

### Philosophical underpinnings

The approach used in the study of the patients’ life situation is inspired by phenomenology and hermeneutics [21, 22]. Phenomenology is seen in this study as an epistemological stance for exploring first-person accounts of what it is like to live with oesophageal cancer. The starting point is about how phenomena are experienced pre-reflexively, namely experiences from the patient’s lifeworld [23]. In this project we wish to look at how the patients experience life with an incurable disease; therefore, the focal point is experiences of the illness however, in order to gain deep insight into the meaning of the patients’ experiences, we will combine the phenomenological descriptions with hermeneutic interpretation [24]. In hermeneutics, understanding and interpretation is a fundamental ontological condition for human existence [25]. This means that it is part of life itself that we are in a continuous process of understanding. Hermeneutics are concerned with interpreting the surplus meaning contained in the human life world and recognition takes place via interpretation. The hermeneutic approach is as such involved as a basic condition for understanding, and the basis for interpretation is given by texts produced by human as fixations of expressions of life and reality. Reality in this sense might not only be viewed as a given network of facts but as anticipation of the potential, not yet in existence.

### Recruitment and participants

The study included patients with inoperable oesophageal cancer of the squamous cell carcinoma type. They were recruited from the Department of Cardiothoracic Surgery at Rigshospitalet in Denmark where around 100 patients are treated for incurable oesophageal cancer annually. Participation requirement was the ability to understand and speak Danish. Patients were approached by the first author (LL) and told about the purpose of the study when admitted to the department and were provided with an appointment for the interview. The patients, namely seven women and 10 men aged 54 to 74, took part in individual qualitative interviews from January to October 2017. The interviews were carried out by LL and MM 1 to 23 months after the patients had received a diagnosis. During the study period, none of the interviewers participated in the daily care of the included patients. Additionally none of the researchers were involved in the care provided to potential participants at the time of recruitment. As we were interested in the phenomenon of how incurable oesophageal cancer disrupts the patients’ lives and how the patients experience and adapt to life with the disease, we included both patients who were recently diagnosed as well as those who had lived longer with the disease. The rationale for the difference in time frame after diagnosis was as such to obtain variation [26] in the patients’ experiences of living with the illness according to the purpose of the study. The time frame of 1–23 months after diagnosis was not determined in advance, however this was the greatest variation possible during the study period.

### Ethical considerations

The patients were informed orally and in writing that their data would be treated confidentially and that any form of data that could be linked to the participants would be pseudo-anonymized. They were also told that they could withdraw from the study at any time without any implications for their further treatment. Written consent was obtained. Approval was received from the authorities in the Danish Data Protection Agency under the Capital Region of Denmark: RH-2016-375 and the study were undertaken in accordance with the guidelines of the Danish Ethics Research Committee.

Sensitive recruitment of patients living with incurable illness is important in building relationships and in establishing participation in interviews. Therefore some ethical considerations were of particular importance; time and place for the interviews were chosen at the patients’ convenience; interviews in the hospital setting were conducted in an undisturbed room; and at the end of each interview we provided the opportunity for the patients to ask questions about their hospitalisation and treatment. Both interviewers had backgrounds as clinical nurses in the department and experience in the care and treatment of this patient group and as such had the possibility to answer general questions. Additionally a collaboration agreement between research team and health care team was established in order to consider and manage if any incidents might occur during interviews. If the patient had any urgent physical or emotional problems or needs, an appointment with their health care team could be arranged right after the interview. None of the eligible patients declined to participate in the interviews, and no adverse events occurred during interviews.

### Interviews

Data was collected in qualitative interviews inspired by Kvale and Brinkmann [27]. The purpose of this method was to obtain descriptions from the lifeworld of the interviewees and to use these to interpret the significance of the phenomena that were described. Kvale and Brinkmann describe two different styles of interviews by using the metaphors “minor” and “traveler” [27]. The minor is actively searching for specific knowledge and “digs nuggets of knowledge out of a subject’s pure experiences”, while the traveler opens himself to what he/she might see along the way. He “wanders through the landscape and enters into conversations with the people he/she encounters”. We designed an interview guide from both viewpoints and as such, we were being open towards the unexpected while still keeping focus on the questions to cover the purpose of the study. The semi-structured interview guide contained open questions, allowing further detailed questions about the phenomena described. The interviews touched on the patients’ experiences – how they experienced life with the illness, if and how symptoms and side effects affected their life, any changes in their everyday lives and social relations, any need for support and questions of an existential nature. The interview guide is presented Table 1. The interviews, lasting 30 to 90 min, were recorded and transcribed verbatim.

**Table 1:** Interview guide

Can you tell me about your experiences of:	
▪ Everyday life before illness	
▪ Everyday life with illness	
▪ Troublesome symptoms, thoughts, feelings and how these might affect everyday life	
▪ How the illness affect life	
▪ Social relationships / family	
▪ Problems, needs or difficulties in everyday life	
▪ Hospitalization and treatment	
▪ Interactions with health care professionals	
▪ Thoughts and feelings, hopes and dreams about the future	
▪ Suggestions for support	

### Analysis and interpretation

The method for analysing and interpreting the qualitative interview texts was inspired by Kvale and Brinkmann [27]. They offer a data-driven inductive analysis approach based on phenomenology and hermeneutics. The phenomenological analysis is called meaning condensation and consists of five steps. First, the interviews were read through to form a picture of the whole. Units of meaning were then determined as they emerged from the interviewees. In the third step, the natural units of meaning were reformulated as simply as possible, presenting the statements as themes from the viewpoint of the interviewees and as the researchers understood them. In the fourth step, the units of meaning were questioned based on the specific objectives of the study. In the fifth step, a descriptive statement based on the essential themes was established in order to concretise the essence of the phenomenon of ways in which incurable oesophageal cancer disrupts the patients’ lives and how the patients experience and adapt to life with the disease. Subsequently, the sub-themes that together form the phenomenon of essence were described. A working group of three members; clinical nurse (LL) and two researchers (MNS/MM), analysed the interview texts independently to discuss and thematise the most significant aspects of the patients’ experiences. The analysis and interpretations focused on the impact of the ways in which incurable oesophageal cancer disrupted the patients’ lives and how the patients experienced and adapted to life with the disease. The preliminary results were discussed with all authors.

According to Kvale and Brinkmann interpretation is a prerequisite when drawing meanings from the analysed data and attempting to see these in some larger context [27]. Interpretations arose when patterns, themes, and issues were discerned in the data and when these findings were seen in relation to one another and against larger perspectives. Data analysis and interpretation are not a schematic procedure, but rather a circular process in which the researchers worked back and forth in the material. The final analysis and interpretations were discussed with all authors to critical interpret and agree upon the findings. The findings from the interviews are presented as one essential theme and four subthemes extracted from the phenomenological meaning condensation and the subsequent interpretations.

## Results

In its essence, the analysis and interpretation reveal how patients with incurable oesophageal cancer experience metaphorically to end up at a “table in the corner”. The illness is controlling the patients’ everyday lives while the patients are left alone with existential thoughts on the future. The patients expose a lack of continuity during the course of the illness and treatment and are experiencing loss of identity, community and dignity.

In the following, we zoom in on four sub-themes that illuminate the ways in which incurable oesophageal cancer disrupts the patients’ lives and how the patients experience and adapt to life with the disease: “sense of isolation”; “being in a zombie-like state”; “one day at a time”; and “at sea”.

### “Sense of isolation”

#### Eating difficulties forces the patients to withdrawal from social interactions

The illness affects the patients’ social interaction, especially social interaction around mealtimes. Some patients feel embarrassed and undignified when eating in the company of others, and they often end up withdrawing from social situations: “The best thing about Christmas Eve is the roast duck. But I gave it a miss. I didn’t even feel like visiting my son and celebrating Christmas with him. I decided to stay home”. “Sometimes I can’t eat food at all, and then I have to throw the food up again, and it’s not nice - and not at all when you are with others - it takes some of your dignity, I think”. As their illness progresses, the patients increasingly isolate themselves, some patients, even more, when fed through a tube. The feeding tube becomes a symbol of their illness and one patient report that her grandchildren did not want to sit next to her after she had been given a feeding tube. Others avoid social situations altogether, one patient commenting that he specifically requests a table in the corner when he is in public places.

The patients do not feel up to taking part in family occasions like they used to. Nor do they feel up to taking an interest in their children or grandchildren’s lives and this makes them feel, in their own words, hurt and upset. Furthermore, it can be hard to share these thoughts, emotions, and worries: “I don’t share my thoughts with my husband. We’re worn out. He can’t take any more. I don’t really have anyone to talk to”. Others report not feeling able to share their thoughts with either relatives or healthcare professionals. Some patients cannot cope with their relatives being emotional or expressing their worry or concern and so choose not to involve their social network more than is necessary. Some patients, however, say that it has done them good to share their thoughts with the medical staff. Health care professionals might as such be an important stand-in in the patients’ social life with the patients finding it hard to share their emotions and worries within their usual social relations.

### “Being in a zombie-like state”

#### Loss of control and confidence in own body caused by symptoms and treatment

The patients’ physical symptoms are interfering and controlling factors in their daily lives. The patients talk of how pain relief treatment makes them tired and dulls their senses so that they feel like they are “in a zombie-like state”. Because of these side effects the patients sometimes turn down pain relief medication, which affects their mood and enjoyment of life: “My daughters have told me I’ve become a cross old woman. It’s because I’m in pain. I’m not putting on an act – I look cross because it really hurts”. The patients also feel alone and left at a “table in the corner” when struggling and coping with the pain.

Pain and other physical symptoms such as increased production of mucus coupled with a recommended elevated sleeping posture mean discomfort and sleeping problems. The patients start to feel tired and exhausted in their day-to-day lives and describe how they metaphorically experience walking around like “zombies” and how ordinary, everyday chores, such as cleaning, cooking and gardening seem insurmountable. Consequently, they have to ask others for help, which is not always easy, and the patients perceive being a burden to others resulting in loss of dignity and a change in their own identity: “My energy has completely disappeared, so it is hard to get started at home, and my wife has to do everything. I have always been working and it was me doing the gardening, but now … Now it is hard to get dressed. I’m very tired, and I can’t do the same as before, and it takes some of my dignity”, “Things have moved fast. Things don’t work anymore. I’m in a lot of pain. I’m limp and I’m dull. I don’t know what to do, because I can’t do anything. It is my wife who does the shopping and arranges things. I’m just doing what I’m told. I’m going out with the garbage. It does something to the dignity, when I cannot be the one I usually was”.

Owing to difficulties in swallowing, many patients also lose a great deal of weight, which changes their appearance and can be worrying and upsetting: “I’ve lost 20 kg since … I’m not sure when. I haven’t been weighing myself because I’ve noticed it from my clothes. One morning I looked at myself in the mirror and thought, there’s something wrong. So, I bought a scale and when I weighed myself I was shocked”. Weight loss affects patients’ identity through impacting on body image and their everyday functioning resulting in loss of control and confidence in their body which adds to the feeling of being in a “zombie-like state”: “After I got the stent [in the oesophagus], I started coughing a lot, and it really hurts and I can’t eat anything. Usually it starts at night which means that I don’t get any sleep. So, of cause I’m tired all the time. I feel like a zombie or whatever, you know slipping into a sleep and then wake up and then fall asleep again. And when you are in pain and when you don’t sleep at night … , then there isn’t much dignity left”. Mealtimes are for the patients a struggle – a struggle for survival. They become irritable and their daily rhythm becomes disrupted to such an extent that it becomes difficult to be an agent in own life and patients might feel like a shadow of themselves banished to “a table in the corner”. To be in a “zombie-like state” carries a symbolic conveyance of patients being hit by activity loss and being homeless in their own body; it describes the interruption of everyday life caused by pain, exhaustion and illness. The “zombie-like state” is an aesthetic for a deep despair in the patients.

### “One day at a time”

#### Clinging to life by keeping things as normal as possible and focus on the present

Living with oesophageal cancer means living 1 day at a time and focusing on the present for the patients in this study. To maintain usual daily routines and living day to day are a way of clinging to life and, therefore, not relinquishing what they know and who they are. In parallel, the patients are also reflecting on life and death. They feel life-threatened, and for some this might lead to a state where they are not able to act: “You have to get on with your life, which can be difficult when you more or less don’t know anymore who you are, when you’ll be here and when you suddenly won’t be here anymore. It’s a different mindset from the mindset you had 10 years ago before you were diagnosed”. Many patients talk about not having much time left to live, but at the same time, they describe their treatment trajectory as if it might provide a cure rather than just relief. As described earlier physical symptoms dominate the patients’ outlook on life and everyday existence, and often they let their symptoms determine the agenda. As such, activities which the patients previously could be engaged in, seems more difficult because of the illness, and there is a disruption between the patients’ definition of himself/herself with regard to the past, the present and the anticipated future. The structures in everyday life become disjointed and the planning horizon, which the patients previously have had, shrinks leaving the patients at a “table in the corner”: “I’ve lost the courage of my life. I’m just sitting and sitting all day, and I’ve never done that before. And the family … , I don’t see them very often anymore. It really makes me sad. And my daughters have told me that I’ve really changed. The future is pretty uncertain which make it hard to plan anything”.

Common to all patients is also a reflection on their own existence and how they should spend the last part of their lives. They oscillate between acceptance of death and not feeling ready, anxiety and ambivalence taking up a large part of everyday life. The patients describe existential fear as a facet of living with the illness: “I get frightened at night and I daren’t fall asleep. Don’t ask me why because if I think about it rationally it’s like, so you fall asleep and don’t wake up – what’s the big deal. However, fear’s just there”. The patients are questioning if their life has meaning, purpose, or value because there are limits or boundaries on it. Despite the existential questioning, the patients approach their incurable illness in different ways. All of them do however express that they live “one day at a time”: “I’ve decided to take things one day at a time. I’m always aware that I might die from my illness, in fact, I very probably will. Things don’t look good, but for me, it’s about getting through this time as best as possible and about me being able to make choices”. The patients want to keep things as normal as possible for as long as they can, taking 1 day at a time and focusing on the present.

### “At sea”

#### The challenge of managing one’s own illness when continuity is lacking

Patients in this study experience a lack of continuity during their treatment leaving the patients with a feeling of being left at a “table in the corner”. They describe how frustrating it is to meet a new doctor each time and to explain the details of their illness all over again. This lack of continuity and constant repetition of their medical history is wearing, and they explain how burdensome it is to “be a talking medical record”. The analysis illustrates how patients feel “abandoned and at sea”, unsure of whom to contact if their symptoms increase. It is challenging for them to manage and coordinate their own illness: “When I started getting worse, I thought, Who should I contact? If you rang up, anyone could pick up the phone. That’s what it felt like to me, anyway. It felt like there was no-one specific to refer to, someone who knew my case. I felt abandoned – at sea. There was no-one to talk to”. Some of the issues that were described by the patients when continuity was lacking include: difficulty getting to appointments and navigating the system; health care professionals who do not understand their situation; and lack of support and symptom management. Consequently, patients feel “at sea” in the encounter with the health care system.

The patients feel that their treatment programme has been put together based on the average patient and fails to take into account their individual situation and needs and the patients are left at a “table in the corner”. From the patients’ point of view, more continuity would be reassuring: “I’ve felt totally abandoned or left to myself. The oesophagus is divided into three, when you come to the hospital, you know, three different departments. It makes communication and coordination difficult. Nobody knows my story and I myself have to be the coordinator of everything. I don’t think that it is about caring for ill people, and I’ve felt it’s been unworthy”. One patient describes how, after going through an extensive programme without having a continuous contact person at the hospital, she was assigned a permanent doctor: “One person who managed my case in a team with two nurses. And a direct telephone number. It’s been a great help. Now I’d get active if I noticed something myself and thought that something needed to be looked at”. The patients found the continuity valuable and important, and when they experience continuity in the relationships with health care professionals they feel having more control over their situation.

## Discussion

This study is one of the first studies investigating, from the patients’ perspective, how oesophageal cancer has an impact and grip on their lives and day-to-day activities. Symptoms of the illness and side effects from the treatment control the patients’ lives and bodies, hindering them from carrying out ordinary, everyday tasks. In the following the four identified sub-themes will be discussed in relation to other research with the purpose of reaching a new understanding of the possible dimensions of the patients’ experiences with their illness. It is an understanding process, in which theoretical or empirical perspectives are drawn on to help clarify and understand phenomena in the patients’ lives.

### “Sense of isolation”

The patients recounted how they felt undignified eating in the company of others, and how their lack of energy caused them to withdraw from social activities and steadily isolate themselves as the illness advanced. The patients also explained the difficulty of sharing their thoughts with others. Milberg and Strang [28] have demonstrated how it is particularly important for people with an incurable illness to have a trusting relationship with their family, which can also reduce feelings of helplessness among family members. Further studies have shown that social relations are important support for patients with incurable oesophageal cancer, although this support can take a variety of forms [14]. However, the patients in the present study felt being a burden to others when needing help to manage everyday tasks. The feeling of being a burden to others may according to Chochinov reflect an assumption or fear of abandonment [29]. In such situations the patient needs assurance that he or she is worthy of attention and support; otherwise, the patient’s sense of dignity can be affected.

In this study the patients experienced loss when it came to mealtimes: loss of the ability to eat, as well as loss of life in that not being able to eat can be perceived as a symbol of life coming to an end [30]. The patients also experienced loss of community, as mealtimes in Western culture are often associated with a time and place for coming together. The patients’ withdrawal from their community could be interpreted as denial or refusal to confront the changes they are facing [31]. In fact, withdrawal gave them a respite from the pain caused by loss, a facet of incurable illness that is also seen in other studies. Dalgaard [32] describes how patients with incurable cancer try to squeeze the most out of life into what’s left of it, which can be mistaken for a defence mechanism such as denial. Being kept free of sorrow is seen as an important element in the grief process, and a review by Strobe et al. [33] concludes that there is no evidence to support the theory that emotional disclosure helps the patient adapt to loss. This finding is backed by other research showing that patients deal with crisis and loss in different ways [34].

Accompanied by difficulties in eating was weight loss which affected the patients’ body image and identity. The patients described a loss of control and confidence in own body. According to Merleau-Ponty [35], the relationship between body and self is unified, meaning that the body is an aspect of the self. In this light, it can be understood how the patients possibilities to be an agent in own life are altered when control of own body is lost. Such awareness is essential for health care professionals when planning treatment and care.

### “Being in a zombie-like state”

The patients described being in a “zombie-like state”. The interviews showed how pain and other physical symptoms started severely affecting the patients’ lives and everyday activities, but also how pain relief treatment made them tired and dulled their senses. Other studies have also reported on the dilemma and the challenges associated with providing pain relief without undesirable side effects [36, 37]. In a systematic review by Pringle et al. [38] pain consistently stood out as being the greatest threat to the patients’ dignity. Studies also show that failure to reduce cancer patients’ symptoms has a significant effect on how they manage their illness and how they experience their own sense of dignity [29, 39]. It is therefore imperative that healthcare professionals do not ignore the impact of symptoms on the patients’ lives and day-to-day activities, as this can lead to lack of dignity in care and treatment. A number of randomized trials reveals that integrating palliative care at an early stage within a general palliative hospital setting significantly improves quality of life, symptom burden, mood, illness understanding, and quality of care for patients with advanced cancer [40–43]. Estacio et al. [44] furthermore conclude that greater awareness of symptom meaning and its influence may facilitate health care professionals exploring symptom meaning more with patients. Thus, healthcare professionals need to be adequately trained in their efforts to provide symptom relief [45].

### “One day at a time”

The analysis of the patients’ accounts in this study illustrated how difficult it was for them to establish a daily life consisting of habits and routines [46] when their life was threatened. During the interviews, patients oscillated between accepting and talking about death as imminent, and therefore living from day to day, and talking about their treatment programme as if it offered the prospect of a cure. Thus, there was alternation between a loss-oriented process and a restoration-oriented process of the dual process model in the way they managed the emotions that arose due to their illness. As a result, we need an approach to patients that can encompass this emotional oscillation. Deterioration in health and quality of life is often rapid within this patient group [2], which highlights the urgency of planning end-of-life care for these patients and discussing the future.

Across the spectrum of different settings, countries, health systems and access to patient care and treatment, palliative healthcare professionals agree on core values. We need a holistic, individualised approach that furthers the patients’ autonomy and dignity [38]. There are models and approaches that could be useful in this context, but it is important that healthcare professionals are given the right education and training to ensure dignified, person-centred care and treatment. Several studies have found that within palliative care, Chochinov’s dignity model can help to ensure individualised, dignified care [47–49]. The model represents the first time that researchers have tried to thoroughly study the concept of dignity from the vantage point of dying patients themselves. Every theme in the dignity model has a clinical correlate, suggesting an area of attention that might help mitigate suffering for patients in palliative care [29]. As such the dignity model can inform clinical decisions and define dignity-concerning pathways for patient receiving palliative care for oesophageal cancer. Furthermore, Missel and Birkelund [14] have also shown how being there and talking can help the patients to self-reflect and thus give them a break from emotionally stressful situations.

### “At sea”

The patients in this study recounted how a lack of continuity during the period of diagnosis and treatment made them feel insecure and lost. They found the lack of continuity exhausting, challenging and a psychological strain, associating it with loss of control over their own situation. Feeling “at sea” and losing control over their own situation increased their vulnerability. Sellman [50] illustrates how an incurable illness can render patients defenceless, destroying their own potential to reduce or minimise their vulnerability. Several studies have shown how patients’ vulnerability can be exacerbated when they are hospitalised [51–53]. Other studies point out the importance of ensuring consistency when it comes to the healthcare staff involved in the treatment. This helps the patients to cope not only with their emotional, existential and practical concerns but also the coordination between the treating institutions and the control and relief of symptoms and side effects. A study by Opstelten et al. [54] demonstrated that continuity in palliative approaches of patients with incurable oesophageal cancer varies widely and findings suggest a lack of guidance for the patients. Furthermore, the authors highlight the need for more evidence on palliative care strategies for oesophageal cancer.

#### Clinical implications

This study has added to the understanding of the experiences of patients living with incurable oesophageal cancer during their palliative treatment. The findings of the study might assist in devising useful interventions that can be tailored to target the need for support for the patients. These patients have a particular need for healthcare professionals who are dedicated to identifying what can be done to control physical symptoms, preserve dignity and provide care, as well as emotional and practical support. As health care professionals we need a holistic individualised approach that furthers the patients’ autonomy and dignity and help the patients to reflect on and planning of end-of-life care. To lessen the patients’ loss of dignity, identity and community, we propose using components such as early detection of palliative needs and providing assigned staff with whom the patients have consistent contact and follow-up contact within a dignity therapy model.

#### Methodological considerations

This study is one of a few qualitative studies illuminating the ways in which incurable oesophageal cancer disrupts the patients’ lives. The qualitative interview method provided insight into patient perspectives and illuminated the meaning of life with the illness. One potential study limitation is the generous timespan of 1–23 months, stretching between the time of diagnosis and the interview, means that a certain degree of variation is to be expected in how the patients live with, cope with and experience having an incurable illness and what challenges they experience as being invalidating. On the other hand this study address the meaning of living with the illness across a quite long period of time meaning that the identified themes may occur both when patients have lived for shorter or longer time with a diagnosis of incurable oesophageal cancer. However, in order to follow the patients’ process of adapting to life with the illness it would have been preferable to conduct a longitudinal study. Trustworthiness [27, 55] of the findings and interpretations was ensured due to the in-depth data collection and prolonged engagement with the data which is considered a strength of the study. Another strength is that to illustrate the themes we used the patients’ own words which also add to the trustworthiness of the study. To ensure transparency and to support the findings of the study the patients are quoted. The incidence of oesophageal cancer is approximately three times higher among men than women. The study included seven women and 10 men, which the reader should bear in mind as far as the transferability of the findings is concerned.

The study included vulnerable patients with incurable illness which could be seen as objectionable and inconsiderate of the patients’ integrity in a distressed life situation and could as such be considered an ethical limitation. However, we experienced that the patients appreciated participating in the interviews and this suggested that we did not cause them any harm. It might be surprising that all of the eligible patients were willing to participate in the study and that they were able to tell their stories of living with incurable illness. We interpreted the patients’ willingness to participate in the interviews as a need for them to talk about their difficult life situation.

## Conclusion

The study has illuminated how the lives of patients living with incurable oesophageal cancer are controlled by symptoms of their illness and side effects from their treatment. The patients’ lives are turned upside down, and they experience loss of health, function and familiar, everyday habits. The patients isolate themselves from their social network as the illness progresses and the symptoms and side effects increase. Furthermore, the patients experience loneliness and a lack of continuity during their treatment, which they find challenging and a psychological strain. Thus, as far as normal everyday life, social networks and the health care system are concerned, the patients metaphorically end up at a “table in the corner”.

## Data Availability

The first author (LL) and the corresponding author (MM) have full control of all primary raw data (interview transcripts) and allow the journal to review our data if requested. All raw data are written in Danish. Data are stored in a locked file cabinet in a locked room at the Copenhagen University Hospital as requested by the Danish Data Protection Agency.

## Abbreviation

SCC: Squamous cell carcinoma
